# Online Social Engagement by Cancer Patients: A Clinic-Based Patient Survey

**DOI:** 10.2196/cancer.5785

**Published:** 2016-08-19

**Authors:** Lawrence C An, Lauren Wallner, Matthias Alexander Kirch

**Affiliations:** ^1^ Center for Health Communications Research University of Michigan Ann Arbor, MI United States; ^2^ Cancer Surveillance and Outcomes Research Team University of Michigan Ann Arbor, MI United States

**Keywords:** cancer patients, Internet, information, social support

## Abstract

**Background:**

The Internet is commonly used as a source of health information, but little is known about the Internet practices specific to cancer patients.

**Objective:**

To understand cancer patients’ use of the Internet as an informational resource and for social support.

**Methods:**

The researchers conducted a survey of 1282 patients at a comprehensive cancer center to assess frequency of Internet access and online behaviors.

**Results:**

Of the cancer patients surveyed, 1096 (85.49%) had Internet access; of those with Internet access, 953 (86.95%) reported going online at least weekly, and 747 (68.16%) reported daily online activity. Grouping Internet users by their level of online social engagement revealed that out of 1096 users, 331 (30.20%) had not sought out social connections online, 227 (20.71%) had read about experiences from other cancer patients, 410 (37.41%) had also written about their personal experiences, and 128 (11.68%) had participated in a formal online group for cancer patients. Increased online social engagement was associated with an increased perception that the Internet was useful for social support.

**Conclusions:**

Internet use among cancer patients was common, and most patients reported that they found useful information about their cancer diagnosis online. Cancer patients who actively posted or shared content perceived more social support from the Internet than those who used the Internet solely as an informational resource or to read about other cancer patients’ experiences. Physicians have a great opportunity to direct users to quality health information on the Web.

## Introduction

In the past decades, there has been rapid growth in the use of the Internet among US adults. The Pew Internet and American Life Project found that 87% of adults have Internet access [1]. Using the Internet to search for health information is common among adult Internet users. The Pew Research Center also found that during the past 12 months, 80% of online adults searched for health information, 26% of people reported reading about or watching another person’s health experience, and 16% went online to connect with others who had the same condition, including 4.6% who took part in an online support group [2]. Patients themselves are not the only ones searching for health information online. Half of the information searches reported were done on behalf of someone else [3].

Cancer patients represent a growing proportion of health information seekers. There are 14.5 million cancer survivors as of 2014, and the current 5-year survival rate is 68%, up from 49% in the 1970s [4]. Data from the Health Information National Trends Survey (HINTS) showed that 81% of the cancer survivors had searched for information about cancer [5]. Younger individuals and those with higher levels of education were more likely to use the Internet as their first source of information, rather than their doctor. The National Cancer Institute also reported that up to 55% of cancer information seekers looked to the Internet first [6]. Other studies have found differing rates of Internet use for cancer prevention information seeking [7], as well as differing information needs by demographic and cancer-related characteristics [2,8].

While online searches for cancer information are common, less is known about cancer patients’ familiarity with, and trust of, the Internet or how patients with different cancers differentially seek information on the Internet. People with serious health conditions may be more or less likely than the general public to turn to online resources. For example, a recent study by the Pew Research Center about chronic disease found that adults with chronic disease report lower rates of Internet use: 62% versus 81% in the general population. However, those people with chronic disease who go online are more likely to participate in online discussions and write blogs [9]. Recent studies of online forums for cancer patients have found that these resources can provide valuable emotional and social support for patients [10-15].

Online communities are popular and show promise for meeting cancer patients’ needs for information and social support, but there is little known about patients’ real-life experiences with a range of websites that can offer online communities [16], or how these online mechanisms can be used to improve social support for cancer patients [17]. This study aims to describe cancer patients’ use of the Internet and, in particular, their engagement with online social activities related to their cancer diagnosis and treatment.

## Methods

### Survey Development and Study Population

A team of physicians, oncologists, cancer nurses, and communication/health literacy experts at the University of Michigan Comprehensive Cancer Center (UMCCC) developed a patient survey to assess a range of behaviors and health experiences of patients at the cancer center, including use of computers and the Internet, information and social support needs, basic demographic and health information, and quality of life. Questions for the survey were adapted from publically available instruments, including the Pew Internet and American Life Survey [18] and the 2007 Health Information National Trends Survey [19]. The survey was reviewed and approved by behavioral health specialists, a patient advocacy group, and the cancer center administration.

Study staff approached all patients present at seven of the UMCCC clinics during a 2-week period from August 23, 2010, to September 3, 2010. Patients were asked at appointment check-in whether they would like to participate in the study. The paper-and-pencil survey took participants an average of 15 minutes to complete. Participation in the study was voluntary; however, if individuals agreed to participate, they were provided with a US $2 incentive coupon to redeem at food services vendors within the hospital. Participation was also anonymous; no personal identifying information was collected from the patients. Paper survey responses were double entered and coded by a third-party vendor. This study was determined exempt by the Institutional Review Boards of the University of Michigan Medical School (HUM00039172).

### Derived Measures

The independent variable used in this analysis was *level of online social engagement*; this was defined as the type of social interactions the participant reported online, was measured using questions about specific online activities related to health, and was adapted from the 2007 HINTS [19]. Internet users were categorized into four exclusive groups by their reported level of online social engagement. Those in the first group report *no social engagement*, such as reading about other patients or sharing their own experiences. The second group is comprised of *consumers* who read about other patients’ experiences but do not share their own. The third group is *producers*, those who write about their own experiences as cancer patients and share with others. The final group is made up of individuals who participate in a *formal online group* related to their health diagnosis. Table 1 shows the full wording of all questions related to online social activities and this study’s social engagement classification strategy.

Dependent variables for this analysis were related to the perceptions of Internet users. These included (1) usefulness of Internet for cancer-related health information, (2) usefulness of Internet for cancer-related emotional or social support, and (3) positive and negative Internet experiences. Usefulness questions were adapted from the HINTS [19]. The first two items asked patients, “How useful was the cancer-related information you got from the Internet?” and “How useful was the Internet in helping you get encouragement or emotional support (from family, friends, or others) in dealing with cancer or cancer treatment?”

**Table 1:** Levels of social engagement characterized by self-reported participation in Internet-based health activities.^a^

Internet-based health activities	No social engagement	Social consumers	Social producers	Formal group
Read or learned about other patients' health experiences?	No	Yes	Yes or no	Yes or no
Wrote about or shared your own health experiences with other patients?	No	No	Yes to this one, or to one of next two items	Yes or no
Written or posted updates for family or friends about your health or how you are feeling?	No	No	Yes to this one, or the item above or below	Yes or no
Wrote in an online diary or blog?	No	No	Yes to this one, or to one of the above two items	Yes or no
Participated in an online support group or community for people with cancer?	No	No	No	Yes

^a^Participants were asked to respond to the following: “Below are some ways people use the Internet. Some people have done these things, but others have not. Please tell us whether or not you have done each of these things while using the Internet.”

Patients reported reactions to their most recent online search for cancer information using questions adapted from the Pew Internet survey [18]. Patients were asked the following:

Think about the LAST time you searched for information about cancer or cancer treatments. At any point, did you feel: OVERWHELMED by the amount of information you found online; EAGER to share your new health or medical knowledge with others; CONFUSED by the information you found online; RELIEVED or COMFORTED by the information you found online; FRUSTRATED by a lack of information or an inability to find what you were looking for online; CONFIDENT to raise new questions or concerns about a health issue with your doctor; FRIGHTENED by the serious or graphic nature of the information you found online; REASSURED that you could make appropriate health care decisions.

### Statistical Analyses

The distribution of demographic characteristics by levels of online social engagement were compared using chi-square tests for association. Because the survey was administered as a paper-and-pencil survey, there was some item nonresponse, especially among demographic variables. The primary analyses compared Internet usefulness and positive and negative experiences by level of online social engagement. The percentage of patients reporting the Internet as *somewhat* or *very useful*, as well as the 95% confidence intervals, was analyzed using chi-square analysis to determine whether the groups were significantly different in their ratings of Internet usefulness. In addition, Wilcoxon signed-rank tests were used to estimate if there were significant differences between ratings for information and social support usefulness. Comparison of usefulness ratings between levels of social engagement, with *no social engagement* as the reference group, was reported using unadjusted logistic regression.

The distribution of positive and negative experiences reported by cancer center patients were evaluated by first assessing each individual item across groups using chi-square tests for each individual item. Then two summary variables were created to represent the total number of positive and negative experiences by person. We then estimated the overall mean, as well as mean by level of social engagement, and used the Wilcoxon signed-rank test to assess whether significant differences between numbers of positive and negative experiences existed. Differences in the mean numbers of positive and negative experiences between groups were estimated using unadjusted ordered logistic regression. Finally, a variable representing the difference between the number of positive and negative experiences was analyzed using a one-way analysis of variance (ANOVA) to test the differences by level of social engagement. All statistical analyses were completed using Stata version 13.1 (StataCorp).

## Results

### Patient Characteristics

Table 2 summarizes the demographic and health-related information of this sample. The sample size for this study was 1282 patients, which represents a 75.01% (1282/1709) response rate of all scheduled patient visits during the 2-week survey period. Item nonresponse was low overall; the variables with the highest percentage of missing values were *years since cancer diagnosis* (87/1282, 6.79% missing) and *age* (40/1282, 3.12% missing). The majority of patients interviewed were female (768/1282, 59.91%), white (1133/1282, 88.38%), and over 50 years old (922/1282, 71.92%). Patients were highly educated; 44.77% (574/1282) had a college degree. The most common cancer diagnoses were leukemia/lymphoma (326/1282, 25.43%) and breast cancer (298/1282, 23.24%), which is representative of the patients at this center. About half of the patients were diagnosed with cancer in the past 2 years (613/1282, 47.82%), 72.23% (926/1282) reported at least one other major chronic health condition, and 35.26% (452/1282) of participants reported their health as fair or poor (see Table 2).

**Table 2:** Demographic and health data of the survey sample (N=1282^a^).

Characteristic		n (%)
**Gender**	Male	512 (39.94)
	Female	768 (59.91)
**Age in years**	<50	320 (24.96)
	50-69	701 (54.68)
	70+	221 (17.24)
**Race**	White	1133 (88.38)
	Nonwhite	142 (11.08)
**Education**	High school or less	288 (22.46)
	Some college	407 (31.75)
	4-year degree or higher	574 (44.77)
**Years since cancer diagnosis**	<1	270 (21.06)
	1-2	343 (26.76)
	3-9	373 (29.10)
	10+	209 (16.30)
**Cancer site^b^**	Leukemia/lymphoma	326 (25.43)
	Breast	298 (23.24)
	Cutaneous	176 (13.73)
	Prostate/urological	173 (13.49)
	Gynecological	161 (12.56)
	Gastrointestinal	144 (11.23)
	Sarcoma/soft tissue	80 (6.24)
	Thoracic	46 (3.59)
	Head and neck	41 (3.20)
	Thyroid/endocrine	23 (1.79)
	Neurological	12 (0.94)
	Other/unknown	17 (1.33)
**Comorbid conditions**	Any comorbid conditions	926 (72.23)
**Self-reported health^c^**	Poor	100 (7.80)
	Fair	352 (27.46)
	Good	521 (40.64)
	Very good	215 (16.77)
	Excellent	55 (4.29)

^a^Categories may not add up to the total of 1282 due to item nonresponse on demographic characteristics.

^b^Cancer site is nonexclusive.

^c^Patients were asked, “How would you rate your current health?”

### Internet Use

Table 3 summarizes the computer and Internet use reported by patients. Most (1096/1282, 85.49%) of the respondents reported using the Internet at least occasionally. Internet use was less common for males (427/512, 83.4% males vs 667/768, 86.9% females), people over the age of 70 (142/221, 64.3% 70+ years vs 620/701, 88.5% 50-69 years and 303/320, 94.7% <50 years), and those with a high school education or less (174/288, 60.4% high school or less vs 365/407, 89.7% some college and 546/574, 95.1% 4-year degree or higher). Of Internet users, 86.95% (953/1096) accessed the Internet at least weekly and 68.16% (747/1096) accessed the Internet daily. Almost all Internet users had access through a high-speed or wireless connection (978/1096, 89.23%).

**Table 3:** Internet use and experiences of cancer center patients (N=1282).

Activity	n (%)
Have a home computer	1083 (84.48)
Use the Internet	1096 (85.49)
Daily Internet use	747 (58.27)
Looked for cancer information	862 (67.24)
Read about other patients’ experiences	619 (48.28)
Wrote about own health experiences	234 (18.25)
Participated in an online cancer support group	128 (9.98)
Posted health updates for family or friends	452 (35.26)
Wrote in an online diary or blog	95 (7.41)

Patients reported participating in a range of online activities related to their health and diagnosis. The most common online activities were searching for cancer information (862/1282, 67.24%); searching for information about doctors, hospitals, and treatments (732/1282, 57.10%); and reading about other patients’ experiences (619/1282, 48.28%) (see Table 3).

**Figure 1:**
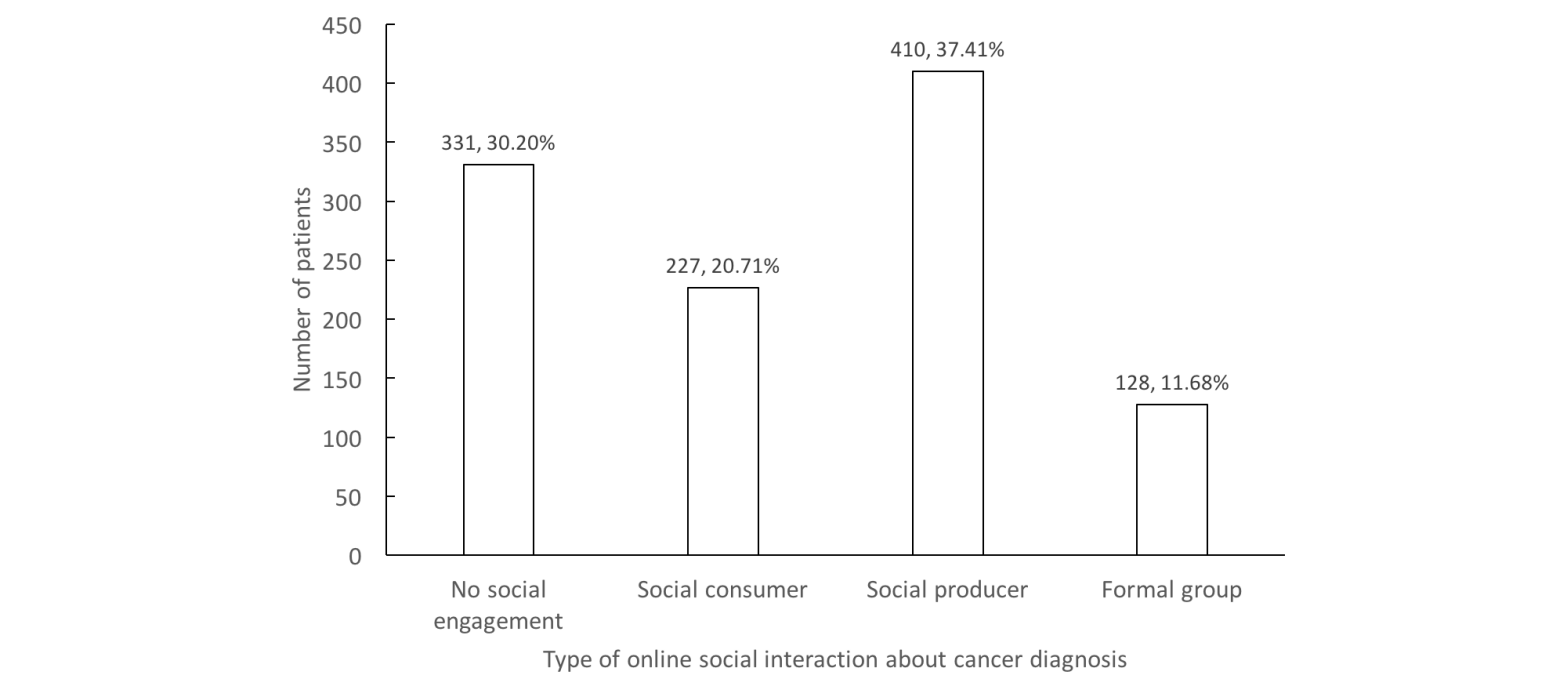
Breakdown of social engagement levels among Internet users (N=1096).

Figure 1 shows the breakdown of Internet users (1096/1282, 85.49%) by their level of online social engagement. Over a third of Internet users (410/1096, 37.41%) were social producers, 30.20% (331/1096) reported no social engagement, 20.71% (227/1096) were social consumers, and 11.68% (128/1096) reported being part of formal online groups (see Figure 1).

Table 4 shows the level of social engagement by key demographic characteristics of the patients. Females (*P*=.003), younger patients (ie, <50 years old) (*P*<.001), and those with more formal education (*P*<.001) were significantly more likely to engage in social interactions online. No other demographic and patient characteristics were associated with level of social engagement, including ethnicity, years since cancer diagnosis, presence of comorbidities, and current health status.

**Figure 2:**
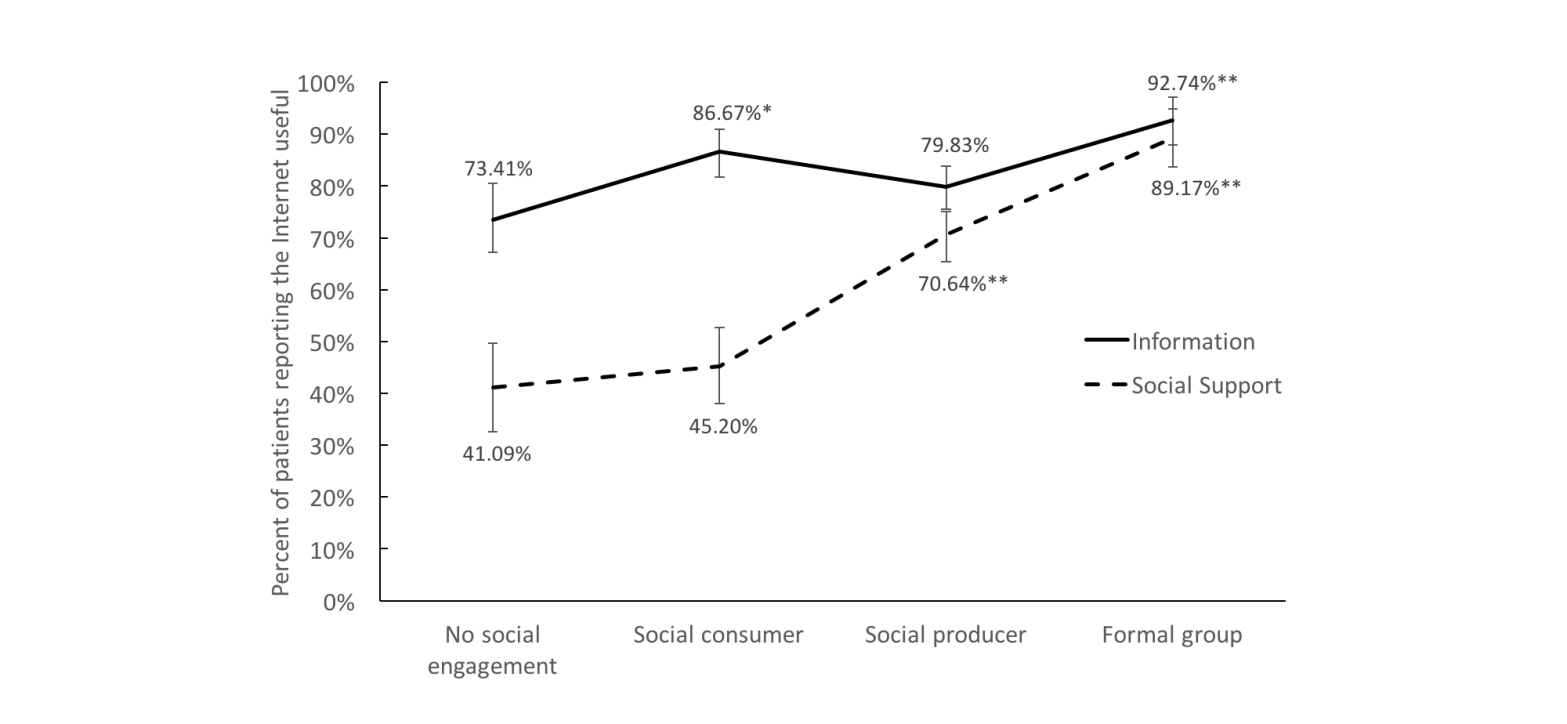
Percentage of patients reporting that the Internet was somewhat or very useful for information about cancer (top line) and social support (bottom line). Bars indicate 95% confidence intervals. Comparisons between the socially engaged groups’ ratings and the “no social engagement” group were made and tested using simple logistic regression. *P=.001, **P<.001.

Figure 2 summarizes patients’ reporting of Internet usefulness by their level of social engagement. Overall, 81.02% (888/1096) of Internet users rated the cancer information they found on the Internet as *somewhat* or *very useful*, and 62.96% (690/1096) of all Internet users reported that the Internet was *somewhat* or *very useful* for providing social support. Ratings of information usefulness were high for participants in all groups, ranging from 73% to 93%. However, social support usefulness was dramatically higher for individuals who were social media producers or who engaged in a formal online group compared to those who reported no social engagement (*P*<.001).

Table 5 shows the percentage of patients by level of social engagement who reported one of the listed positive or negative feelings during their most recent search for cancer information online. Positive and negative experiences were common among patients who reported searching for cancer information online. Only individuals who searched for cancer information were included (913/1096, 83.30%). The most common experiences were feeling confident to raise new questions with their health care provider (546/913, 59.8%) and feeling reassured about making good health care decisions (541/913, 59.3%). There was significant variation by level of social engagement for all experiences. Increasing levels of social engagement were associated with increases in both positive and negative experiences (both *P*<.001).

**Table 4:** Level of social engagement of Internet users by key demographic factors (N=1096).

Characteristic		No social engagement, n (%)	Social consumer, n (%)	Social producer, n (%)	Formal group, n (%)	*P* ^a^
All patients (N=1096^b^)		331 (30.20)	227 (20.17)	410 (37.41)	128 (11.68)	
**Gender**						
	Male (n=427)	152 (35.6)	85 (19.9)	154 (36.1)	36 (8.4)	.003
	Female (n=667)	178 (26.7)	142 (21.3)	255 (38.2)	92 (13.8)	
**Age in years**						
	<50 (n=303)	68 (22.4)	54 (17.8)	122 (40.3)	59 (19.5)	<.001
	50-69 (n=620)	187 (30.2)	146 (23.6)	224 (36.1)	63 (10.2)	
	70+ (n=142)	64 (45.1)	22 (15.5)	52 (36.6)	4 (2.8)	
**Race**						
	White (n=968)	289 (29.9)	194 (20.0)	374 (38.6)	111 (11.5)	.13
	Nonwhite (n=122)	41 (33.6)	31 (25.4)	34 (27.9)	16 (13.1)	
**Education**						
	High school or less (n=174)	89 (51.2)	18 (10.3)	56 (32.2)	11 (6.3)	<.001
	Some college (n=365)	106 (29.0)	79 (21.6)	137 (37.5)	43 (11.8)	
	College degree (n=546)	132 (24.2)	128 (23.4)	213 (39.0)	73 (13.4)	
**Years since cancer diagnosis**						
	<1 (n=234)	80 (34.2)	47 (20.1)	89 (38.0)	18 (7.7)	.15
	1-2.99 (n=294)	83 (28.2)	56 (19.1)	119 (40.5)	36 (12.2)	
	3-9.99 (n=329)	92 (28.0)	79 (24.0)	110 (33.4)	48 (14.6)	
	10+ (n=177)	57 (32.2)	33 (18.6)	69 (39.0)	18 (10.2)	
**Comorbid conditions**						
	None (n=327)	87 (26.6)	77 (23.6)	124 (37.9)	39 (11.9)	.28
	1 or more (n=769)	244 (31.7)	150 (19.5)	286 (37.2)	89 (11.6)	
**Self-reported health**						
	Poor (n=86)	23 (27)	17 (20)	31 (36)	15 (17)	.14
	Fair (n=284)	80 (28.2)	58 (20.4)	115 (40.5)	31 (10.9)	
	Good (n=449)	138 (30.7)	85 (18.9)	172 (38.3)	54 (12.0)	
	Very good (n=197)	56 (28.4)	47 (23.9)	73 (37.1)	21 (10.7)	
	Excellent (n=53)	22 (42)	17 (32)	10 (19)	4 (8)	

^a^*P* values are from chi-square analyses comparing level of social engagement by patient characteristics.

^b^Categories may not add to the total of 1096 due to item nonresponse on demographic characteristics.

**Table 5:** Cancer patients' reports of most recent Internet search for cancer information, among those who reported information searching (N=913).

Feelings reported	No social engagement (n=212), n (%)	Social consumer (n=212), n (%)	Social producer (n=364), n (%)	Formal group (n=125), n (%)	*P*
Overwhelmed	55 (25.9)	83 (39.2)	169 (46.4)	53 (42.4)	<.001
Eager	39 (18.4)	70 (33.0)	148 (40.7)	75 (60.0)	<.001
Confused	38 (17.9)	66 (31.1)	134 (36.8)	49 (39.2)	<.001
Relieved or comforted	45 (21.2)	104 (49.1)	163 (44.8)	87 (69.6)	<.001
Frustrated	23 (10.9)	50 (23.6)	99 (27.2)	44 (35.2)	<.001
Confident	67 (31.6)	141 (66.5)	243 (66.8)	95 (76.0)	<.001
Frightened	33 (15.6)	66 (31.1)	119 (32.7)	44 (35.2)	<.001
Reassured	74 (34.9)	128 (60.4)	236 (64.8)	103 (82.4)	<.001

**Figure 3:**
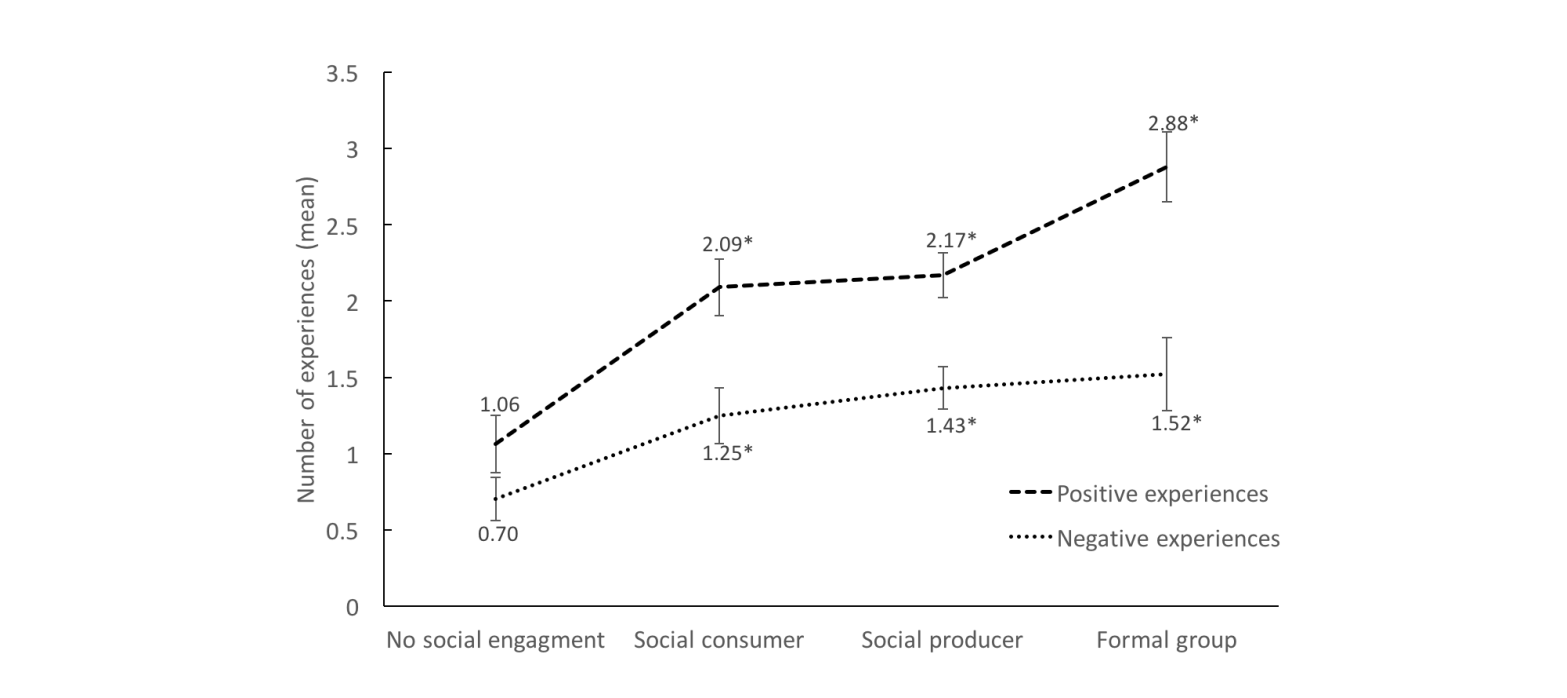
Mean of sum of the positive (top line) and negative (bottom line) experiences of cancer patients searching for cancer information online (n=801). Bars indicate 95% confidence intervals. Comparisons between the socially engaged groups’ ratings compared to the “no social engagement” group were made and tested using ordered logistic regression (ologit). *P<.001.

Figure 3 shows the sum of positive and negative experiences reported by each Internet user by level of social engagement. Positive experiences were higher for all levels of social engagement (Wilcoxon *P*<.001). There was a significant difference between the means by level of social engagement, as individuals who had no social engagement had the smallest difference (0.36 more positive than negative experiences on average), and those participating in formal groups had the largest difference (1.36 more positive experiences) (*P*<.001).

## Discussion

### Principal Findings

Our survey of cancer patients at a comprehensive cancer center found high rates of Internet use (over 80% of patients), including high rates of content production (over 50% of Internet users). Despite the high levels of reading about patients’ experiences and sharing their own personal experiences, very few patients reported being a part of a formal support group.

The vast majority of patients reported that the information about cancer they were able to find on the Internet was useful. However, patients who have written about their own experiences or taken part in a formal group were much more likely to report that the Internet was useful for social support. These findings support the validity of the categorization of social engagement. Patients who have no social engagement or who are solely social consumers were less likely to find social support from their Internet experience. However, social producers and patients engaged in formal support networks reported that the Internet provided them with the greatest social support as well as information about their diagnosis, suggesting that the real social benefits come from sharing personal experiences.

Regardless of the level of social engagement, both positive and negative experiences while online were common for patients. As people reported more social engagement, their numbers of positive and negative experiences also increased. However, there was a greater increase in the number of positive experiences than of negative ones. Overall, patients’ experience of the Internet appeared more positive than negative, and patients who engaged in social support networks online found value in those interactions.

### Limitations

Observations from this survey have a few limitations. The information was collected from a single point in time, and no conclusions can be drawn about a causal effect of online behavior on feelings of social support. The University of Michigan has limited ethnic diversity and a highly educated sample, and the results here may not reflect the larger population of cancer patients and survivors. Finally, there have been rapid changes in the use of technology, especially on mobile devices, in the time since this survey was completed.

Future work should repeat the survey with a larger, more representative sample. This will allow researchers to better understand the population that is using the Internet for social support, and how Internet use varies by age, cancer type, and education. Understanding these differences can inform the development of cancer-specific Web resources that are appropriate for their audiences.

### Comparison With Prior Work

Previous nationally representative studies have reported statistics about use of the Internet for searching for and sharing health information. The 2012 Pew Research Center study found that 26% of Internet users had read about or watched another person’s experience with a health issue, and 16% of people reported seeking out other people with the same condition [3]. A report based on the 2008 Pew Research Center survey found that among patients with chronic diseases, 37% had read about someone else’s experience online, 20% had created their own content related to their health condition, and 7% had participated in an online support group [9]. In this study of UMCCC patients, over half (58%) reported reading about others’ experiences, 22% wrote about their own experiences, and 12% participated in a formal online group related to their health. That these percentages are higher than the national average or even the rates among people with chronic disease is not surprising, given the severity of a cancer diagnosis. These results are also consistent with analyses of the HINTS data, which have shown rising levels of cancer information seeking between 2003 and 2013 [5]. The HINTS data have also shown higher rates of social Internet functions among cancer-connected individuals compared to the general public, up to three times higher for activities like writing in a blog or participating in an online support group [2].

It is also important to consider how the online experiences of cancer patients compare to other health information seekers. Cancer patients in this study rated online information on cancer to be useful (41% *very useful* and 40% *somewhat useful*). This is comparable to results from the HINTS, which found that 46% of online health information seekers rated cancer information to be *very useful* and 43% *somewhat useful* [19]. In contrast, cancer patients’ emotional reaction to online information may differ compared to general health information seekers. In a 2006 report from the Pew Internet Project, people searching for health information generally reported high rates of positive experiences and low rates of negative experiences. The positive emotions included feeling reassured (74%), confident (56%), and relieved (56%). Negative feelings were reported much less frequently; 25% of respondents felt overwhelmed, 22% frustrated, 18% confused, and 10% frightened [20]. Among cancer patients at the University of Michigan, there were similar rates of positive experiences—69% reassured, 68% confident, and 51% relieved—but higher rates of negative feelings—45% overwhelmed, 36% confused, 33% frightened, and 27% frustrated. These higher rates of negative experiences mirror concerns that providers have about the quality of the information that patients access online [21,22].

Other recent studies have confirmed that looking online for health information and support has become the norm for most cancer patients: upwards of 80% [23-25]. Receiving a cancer diagnosis has become a recognized major life event, and patients and families have very high information needs in the weeks following an initial diagnosis [21,26]. The findings from this survey of cancer patients add to the growing evidence for the need for quality online avenues for patients. Despite the increasing dependence on online sources, most patients still consider their doctors to be their primary information source [25]. Rather than considering Internet searches a threat to physician-patient trust, there is evidence that patients who seek out information on their own are more active participants in their own care [22]. Providers may have a great opportunity to help patients by proactively recommending online resources that will provide quality information and support.

### Conclusions

Internet use and health information searches by cancer patients was common in this sample, but there were varying rates of online social engagement among patients. About half of the cancer patients surveyed were social *producers* who posted and shared content about their experiences with cancer. Social producers were most likely to benefit from perceived positive social support via the Internet, and producing content was associated with higher occurrence of positive search experiences. These findings suggest the need for additional research to examine what types of information and messages lead to patients having positive experiences, and how health professionals can help patients avoid negative experiences online.

## Abbreviations

ANOVA: analysis of variance
HINTS: Health Information National Trends Survey
ologit: ordered logistic regression
UMCCC: University of Michigan Comprehensive Cancer Center
